# Effect of Higher Requirement on Medical College Attendance

**Published:** 1917-10

**Authors:** 


### EFFECT OF HIGHER REQUIREMENT ON MEDICAL COLLEGE ATTENDANCE

A short time ago, the present writer had an opportunity to become acquainted with the members of the medical profession in a number of small cities, and he was struck with one singular fact—the scarcity of young graduates. The members of the profession met, usually were advanced in years, most of them having been in practice ten, twenty or more years, while very few had less than five years to their credit. Judging from this fact, and considering the constantly increasing demands made by the public for new services as also the proportion of the recent graduates who go in for research-, laboratory- and other work that takes them out of the line of general practice or even of the specialties, a decided scarcity of doctors must be anticipated within a few years.

For a generation, we have had it dinged into us that the profession is overcrowded. Most of us have realized the truth of this and felt that more than one of our competitors might well be spared and his practice turned over to us. That we should have one doctor to every six hundred of our population, while Russia had but one to six thousand, only emphasized the problem before us; and so, the cry of fewer doctors and better ones was reechoed everywhere, with no dissenters. In fact, it became a shibboleth; and the man who so much as suggested that the demands made by the American citizen upon the medical profession were somewhat in advance of those of the Russian mujik became suspected of lukewarmness.

Under the influence of such universally accepted teaching, the medical course has been advanced steadily, from two years, to six; its cost has increased even more than proportionally; the entrance requirements approximate those of a collegiate degree; and the number of medical schools lessens yearly as the weaker succumb to the squeezing-out process. Moreover, the actual number of students in attendance in all American medical colleges falls rapidly.

Certainly, the grade of the American doctor has been raised, while the cost of professional attendance—to the physician himself—has risen very much more than its quality, without, however, an adequate increase in monetary returns. With the new duties saddled upon us by the medical supervision of school children and by other public health duties, and with the development of this prophylactic movement that may naturally be expected, the result of a greatly lessened supply of graduates, with an increasing population, will not be long in becoming evident.

Broaden our definitions, however, and an entirely different state of affairs confronts us. Instead of basing our argument on the medical profession as enumerated in the standard directories, ask about the members of the healing fraternity as embracing everybody who is at present engaged in treating the sick, and we find that the number of these is, actually and relatively, greater than ever. Osteopaths, Christian Scientists, and the whole brood of outsiders swarm everywhere, until in those states in which the lines delimiting the regular profession are most closely drawn the outsiders actually outnumber legitimate practitioners. Twenty millions of America's citizens rely on these kinds of medical advisers, it is said—and we believe that the number is grossly understated, that forty millions approaches the truth more nearly.

Furthermore, in spite of the pressure brought to bear by the Association of American Medical Colleges and of the increased requirements of the medical state boards—especially since the publication of the Flexner report—there still are numerous medical schools that offer courses leading to the degree of Doctor of Medicine although they are not adequately provided with laboratory equipment and clinical material to turn out physicians of the high type demanded today. Being less expensive than the "Class A" or "Class B" schools, these smaller, inferior institutions naturally attract young men of limited means who want to get their degree as rapidly and cheaply as possible.

And so, the question is answered, Where are the medical students? The young aspirants flock to those schools that make medical education cheap and easy, or to irregular schools of healing. This always has been so—and human nature never changes.

Young men under the direct influence of our best members are taught that only the few should enter medicine, youths who, according to Dawson, can devote $10,000 to the expense of preparation. A man who can afford such a sum for education is not penetrated by a very lively sense of the necessities of life, and, naturally, the scientific side of our multifarious studies appeals to him. The active, alert, wideawake American for whom his chosen lifework must include the earning of a living is likely to take a less academic view of the problem; he looks at the practical side and, consequently, is apt to choose the minor college or even some school of "drugless healing." He gets through with his course years before the other, and by the time young Moneybags secures his diploma young Hustler has built up a lucrative practice.

Do not forget that to the layman, as a rule, a doctor is a doctor, and that one kind of doctor, to him, looks very much like any other. Of whatever school, all offer a legal opportunity to make a living by healing the sick, and, as he sees it, the one kind offers a far easier way than does the other. If, then, the young lay aspirant thinks at all of the relative morality of the two, his judgment is influenced by newspaper talk as to "Medical Trust," unnecessary operations, and such like, and sees no reason for discrimination.

However, this young man does not take into account the fact that the degree of Doctor of Medicine has a significance differing from that of "Doctor of Osteopathy,"

''Doctor Chiropractic,'' ''Doctor of Naturopathy,'' and all the rest of them. It is unfortunate that an academic dignity like that of the doctor degree has fallen into such sad straits, but that is something which we can not alter. We can, however, see to it that the degree of Doctor of Medicine really means something and that its possession conveys a warranty that the holder thereof has given proof of his fitness to act as medical adviser to the sick.

While deprecating absolutely the host of nonmedical cults of healing, we do not mean to suggest that nothing good can come out of small colleges, or that the poor student does not have a chance today to gain a first-class medical education. Indeed, we are convinced that the reverse holds true, and that these schools turn out many well educated and capable graduates. Some of the foremost medical coryphees in our country received their education in schools that, today, would be ranked as belonging to Class C or even D, but they had the stuff out of which physicians are made and forged to the front ranks of their chosen profession in spite of their insufficient education, not because of it. It depends largely upon the student himself whether he will get the best possible results. The student who works his way through a medical school usually is a fighter and may be trusted to be sincere in his efforts to obtain a thorough medical education.

We are convinced that the present policy, namely, that of demanding from medical students a great deal, and now more than ever, and of refusing to admit them to the study of medicine until they shall have accomplished a minimum of two years of college work or its equivalent—is a proper one, and that it bears within it the promise, in the future, of better and more efficient medical care for our population than has been possible in the past. The suspicion with which the medical profession is viewed in many places, the bad odor in which it stands is an inheritance born of sins committed by physicians through centuries. The medical profession must redeem itself by educating the people to an understanding of its better and truer aims, of the wider and the farreaching extent in which physicians desire to exert their influence for the good of mankind.

All this is very good academically; practically it remains a fact that the attainment of a "Class A" medical education has become impossible, except at a financial sacrifice of which but few men and women are capable.

A strange anomaly is presented in our academic institutions. More boys and girls now are going through colleges and universities than ever before in the history of the country; and they are doing so because it is made so easy for them. But, on the other hand, fewer young men and women are passing through the professional schools now than ever before in our history, and this is because it is made so difficult for them, not alone through the many educational demands and requirements, but also because of the present-day excessive expense. It is not altogether a question of scholarship—that of money occupies the foremost place.

We believe that the possibility to secure a professional training, more particularly a medical training, should be made accessible to every ambitious young man and woman. In other words, we believe that high rates of tuition and other financial difficulties should be reduced, so that it may be as easy for a bright young student, financially, to get through a medical school as it is now to get through a state university.

At the present time and under existing conditions, advances in medical requirements are developing a smaller, but more finely trained class of physicians, but at the same time they are responsible for greatly increasing the numbers of practitioners in the irregular classes that do not come under the purview of the state examiners. Let us take heed, lest in our honest desire to improve medical education we work injury, unwittingly, upon the public whom we wish to benefit.—*Clinical Medicine.*

